# Multi-parametric characterization of drug effects on cells

**DOI:** 10.12688/f1000research.26254.2

**Published:** 2021-01-21

**Authors:** Yael Paran, Yuvalal Liron, Sarit Batsir, Nicola Mabjeesh, Benjamin Geiger, Zvi Kam

**Affiliations:** 1 Department of Molecular Cell Biology, The Weizmann Institute of Science, Rehovot, 76100, Israel; 2 IDEA Biomedical Ltd., Rehovot, 76705, Israel; 3 Department of Urology, Tel Aviv Sourasky Medical Center, Tel Aviv, 64239, Israel; 4 Department of Immunology, The Weizmann Institute of Science, Rehovot, 76100, Israel

**Keywords:** Microscope imaging, Multiparameter scoring, Drug effect screening

## Abstract

We present here a novel multi-parametric approach for the characterization of multiple cellular features, using images acquired by high-throughput and high-definition light microscopy. We specifically used this approach for deep and unbiased analysis of the effects of a drug library on five cultured cell lines. The presented method enables the acquisition and analysis of millions of images, of treated and control cells, followed by an automated identification of drugs inducing strong responses, evaluating the median effect concentrations and those cellular properties that are most highly affected by the drug. The tools described here provide standardized quantification of multiple attributes for systems level dissection of complex functions in normal and diseased cells, using multiple perturbations. Such analysis of cells, derived from pathological samples, may help in the diagnosis and follow-up of treatment in patients.

## Introduction

Advanced precision medicine enables the use of genetic and proteomic information for the characterization of disease states, based on the correlation with detailed medical records and specific pathological manifestations ^1– 11^. Indeed, multi-component “omics” profiling can report a large number of components of the genome, transcriptome, proteome, interactome and metabolome (to name a few), and detect even a small fraction of them, indicating significant changes from normal ^12– 16^, yet this fraction may not fully overlap with the currently used repertoire of medical manifestations in complex diseases such as heterogeneous cancers ^17– 26^. We address here the possibility of using multi-parametric characterizations of cellular features for identifying novel signatures of functional cell states, and offer quantitative diagnostic, as well as mechanistic measures, of disease progression or suppression following therapy.

It is widely recognized that meaningful understanding of disease states needs to be achieved in the context of the whole body physiology and tissue functional morphology, yet cell-level functional abnormalities lie at the basis of many pathologies, and may thus be identified by recordable cellular attributes. Developments in quantitative light microscopy, either in two-dimensional or in three-dimensional model settings, has been combined with live cell microscopy and recent adaptation of microfluidics technologies to screening and personalized treatment optimization with primary cells, spheroids, organoids and tissue biopsies ^27– 33^. The difficulty in quantitative characterization of multiple cellular features of biological specimens with diverse morphological and molecular properties creates an urgent need for methodologies that can be standardized, yield strong statistical scores and can be automated for effective application in systems-level biomedicine.

The value of multi-parametric analysis is well recognized in flow cytometry ^34^, and is mandatory when the strategies aiming at functional screening depart from cell reporters for specific drug-target interactions. The application of multi-parametric analysis to cell screening has become a common place. Perlman *et al*. ^35, 36^ have designed a titration-invariant similarity score (TISS) based on multi-parametric doze response matrix for each drug, allowing comparison of drug effects to reflect similarity of mechanisms of action. Classification of patterns displayed by tagged proteins in cells ^37– 43^ is a powerful way to sort sub-cellular localizations, but is bound to cellular responses reflected by the labeled epitopes (e.g. nucleus vs. cytoplasm localization). Tanaka *et al.* ^44^ based their analysis on multi-parameter evaluations, using Principal Components Analysis (PCA) to reduce the number of independent parameters so that the multi-dimensional state vectors of cells treated with drugs could be displayed in 3D plots to differentiate or correlate effects and infer similar mechanisms. Melnick *et al.* ^45^ obtained their multi-parametric data vectors from 35 tyrosine-kinase-activated cellular assays responding to 1400 kinase inhibitors, and used clustering in Euclidian space to sort perturbations similar in action to known drugs. Screens have also been developed to dissect cellular mechanisms ^42, 46^ and identify proteins involved in cellular processes using interference RNA perturbations ^43, 47^. Rather than prior definition of the type of effect (e.g. cell death) or a marker to a specific function, the analysis developed here quantifies drug effects on cell phenotypic and molecular attributes in high dimensionality space.

In this study we have determined the effects of the NCI COMBO drug collection [ 48, Plate number 3948] on five cell lines; the well-established cervical carcinoma-derived HeLa cells and four cell lines derived from bladder cancers ^49^, and used light microscopy-based screening to record multiple cellular responses to the tested drugs. Parameters such as total protein levels, cytoplasmic-nuclear distributions and cell death were analyzed at low magnifications (X10/0.25 objectives) ^50^, while high-definition imaging, providing detailed intracellular data about fine protein localization (e.g. cytoskeletal fibers, sub-cellular organelle morphology, etc.) used higher power objectives (60x/.95), providing multi-dimensional information needed to link drug responses with molecular mechanisms and cellular functions.

In order to be able to read properties of cells and measure as many aspects of system-level response to the treatments, we developed an analysis platform for multi-parametric characterization of cellular features and unbiased scores, based on Mahalanobis distances, for identifying the potency of drugs producing a wide variety of effects. The multi-parametric score is a single value representing the difference from control cells, that contain, however, the complete information on the individual contributions of each of the measured parameters, enabling us to identify those that are mostly affected by each perturbation. Furthermore, the score allows quantification of time-dependencies, examination of cell-line differential responses, and prediction of expected synergy of drug combinations.

## Methods

### Cells

HeLa, and four cell lines derived from bladder cancers (UMUC3, TCCSUP, HT1376 and RT4 ^1^) were obtained from the ATCC. Cell lines were maintained in DMEM supplemented with 10% FBS (Sigma-Aldrich, Rehovot, Israel) and penicillin/streptomycin antibiotics. Cells were grown in polystyrene petri dishes.

### Drugs

Drugs were obtained from the National Cancer Institute (NCI). The COMBO plate number 3948 ^48^ includes 77 compounds, 23 of which are FDA-approved anti-cancer drugs. Mechanisms of action include anti-metabolite activity, tubulin binding, DNA damage, Hsp90 binding, as well as inhibition of topoisomerases, kinases, the proteasome, angiogenesis, ion channels, palmitoylation, phosphodiesterase, cyclooxygenase, and aromatase.

### Sample preparation

Cells were suspended and transferred to 384 well plates (thin plastic bottom, Cat #781091 Greiner-Bio One, D-72636 Frickenhausen) using BioMek FX liquid handling robot (Beckman Coulter, Fullerton, CA 92834) at a density of 1000 cells/well and cultured for one day. Drugs were then added at the ten three-fold dilutions, concentration range: (10-5x10 ^-4^)*GI _50_, (GI _50_ is the concentration of the least sensitive line in the NCI60, see *Extended data:* Table S1 ^51^). Following the specified incubation time with the drugs, cells were fixed, labeled with DAPI for nuclei (Life, Molecular Probes D1306), FITC-Phalloidin for F-Actin (Life, Molecular Probes F432) and indirectly immunolabeled for tubulin (Primary Antitubulin antibodies (SIGMA T6199)) and Cy3-labled secondary antibodies (Alexa Fluor546 –Life, Molecular Probes A11030), and washed by the robot. For drug effect measurement, ten 3-fold dilutions in duplicates were arranged in plate rows, starting from 50 times the median effect concentration specified for the COMBO collection. The transfer order and concentrations from the 96-well COMBO drugs plate into six 384-well plates for each of the 5 cell lines are listed in *Extended data:* Table S1 ^51^.

A total of 16 binary combinations were selected from 20 drugs that showed cellular effects. Matrices with two-fold dilutions in duplicate with drug concentrations up and down from the median effect concentrations, as listed in Table 1, were prepared for two cell lines (TCCSUP & UMUC3). Four such matrices were arranged in one 384-well plate, and rearranged for presentation, including duplicate averaging, as “virtual plates”.

**Table 1.** List of drugs that induced cellular responses. Median-effect values were fitted to the scores in Figure 1 using the equation in the Methods. The table lists D _m_ the column number of half effect for the drug rows marked by arrows in Figure 1. The higher the D _m_ number is, the more effective the drug is at higher dilutions. The corresponding concentration for a specific drug is 10*GI _50_ *3^(1-D _m_/2) (3-fold dilutions in duplicates from initial 50 fold dilution of the COMBO supplied concentration, set as 500 times the drug GI _50_).

Plate [row]	Drug name	HeLa	TCCSUP	UMUC3	HT1376	RT4
1 [I]	Curcurbitacin	7	8	6	-	1
2 [E]	Angiogenesis***	-	1	5	1	-
3 [B]	Miconazole	-	4	5	2	-
3 [E]	Tetrandrine	-	4	2	-	-
3 [J]	Pimozide	-	3	3	-	-
3 [K]	Helenalin	-	9	9	-	-
3 [O]	Curcumin	-	2	4	-	-
4 [H]	Radicinin	4	6	6	5	2
4 [I]	Methdilazine	3	4	4	3	1
4 [M]	Perezone	-	4	8	2	2
4 [O]	Ursolic acid	4	3	4	2	1
5 [G]	Celecoxib	-	3	2	1	-
5 [J]	Rhizoxin	1	2	2	3	2
5 [K]	Brasilin	-	4	5	2	-
5 [L]	Maytansine	-	4	6	2	-
5 [M]	BCNU	2	3	6	-	-
6 [B]	Colchicine	-	4	4	2	2
6 [F]	Vincristine sulfate	-	3	4	-	-
6 [G]	Valinomycin	-	-	2	-	-
6 [L]	Nocodazole	9	11	12	7	1
6 [M]	Paclitaxel (Taxol)	-	1	4	-	-

### Imaging

Plate scanning and multi-color image acquisition was performed by WiScan Argus (Idea-biomedical, Rehovot 76705, Israel; https://idea-bio.com) a fast, high-resolution screening microscope system ^52– 54^. Images were stored locally during the screen, and transferred to 60 TeraBytes storage [NetApp, Sunnyvale, CA 94089] accessible to the analysis workstations via fast local Ethernet for visualization of raw images, interactive optimization of the analysis strategy and tuning of user-controlled parameters (see below). The automated analysis pipeline was then run, accumulating analyzed results to a data base, allowing results display and interpretation.

### Analysis pipeline

In order to process the images, we used a computer cluster (Sun Microsystems), consisting of AMD dual-core computer nodes running Linux RedHat. The software used was WiSoft Minerva (Idea-biomedical).

The analysis scripts pipeline ( *Extended data* ^55^; scripts can be used with any software) combines throughput with modularity. Analysis of different specimens and diverse assays requires a wide range of alternative algorithms and interactive capability to select between them and optimize user-defined parameters before processing the whole data. To achieve such flexibility, we broke the analysis into modular steps categorized by their functionality (Pre-processing, Segmentation and Quantification see *Extended data:* Figure S1 ^51^). Sequences of the analysis modules can be integrated like in a jigsaw puzzle and looped in cycle on all images acquired in an experiment. While such modular structure typically compromises performance, we implemented fast communication of images and data between modules via Shared Memory, achieving processing time as fast as would have been the processing time of an integrated optimized program. Every image is annotated during the acquisition (time, fluorescent color or transmitted light, position inside well, well in plate, plate in whole experiment), and the analyzed data carries these annotations, keeping track of the experimental organization (metadata). Calculation and display of statistics of the analyzed results is therefore directly linked with the experimental design structure. The “Plate GUI” is used to display plate-wide scores, and to interactively show (by a mouse click on a well) the original images and the corresponding numerical and statistical analyzed data. The access to all levels of the data is necessary for rational mining of the TeraBytes of digital information at all levels: original images, montages of image tiles, segmented objects, quantified parameters and statistical profiles. The scoring algorithm used here is based on Mahalanobis distances in multi-parametric space (see *Extended data:* Figure S2 ^51^). The attributes calculated for each cell are listed in *Extended data:* Table S2 ^51^.

### Drug combination effect analysis

Combination matrix scores were fitted using Loewe-additivity and Bliss-independence models ^56– 58^ using the following steps:

I. Fitting D _m_ and s in Chou’s medial effect equation to the single-drug response curve (first rows and columns in the combination matrices):

Af+B=A/[1+(D _m_/D) ^s^]+B 

where: 

f           the fractional effect, here from the Mahalanobis score,

A+B     the measured score amplitude at infinite drug concentration,

B          the score baseline at zero drug concentration.

D _m_       the median effect concentration of the drug

s           the Hill parameter

The parameters A,B were first evaluated from the scores response curve minimum and maximum, D _m_ and s were obtained from the linearized equation for the log of normalized fractional effect data: log(1/f-1)=s*log(D _m_/D)

The four parameters A,B,D _m_,s were then used as initial estimates for a non-linear Marquet-Levenberg fit.

II. Using the parameters s _1,2 _for each of the two drugs to “span” the combination effect matrix, f _comb_, as a function of the two concentrations, D _1,2_, and solving f _comb_ from Loewe additivity equation:

D _1_/[f _comb_/(1-f _comb_) ^1/s1^] + D _2_/[f _comb_/(1-f _comb_) ^1/s2^] = 1

III. Using the medial effect parameters D _m1,2_,s _1,2 _ for each of the two drugs to “span” the combination effect matrix, f _comb_, assuming Bliss independence:

f _comb_=f _1_*f _2_=1/[1+(D _m1_/D _1_) ^s1^] /[1+(D _m2_/D _2_) ^s2^]

IV. Fitting the four parameters D _m1,2_,s _1,2 _ using all the values in the combination matrix simultaneously, based on Loewe or Bliss models.

## Results

**Figure 1.**
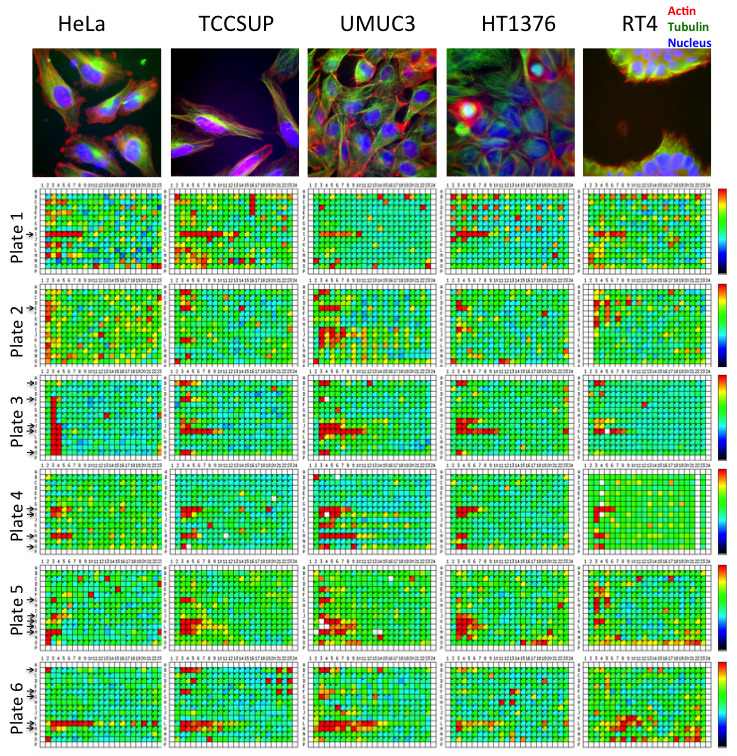
Cell imaging and multi-parameter analysis. Five cell lines were cultured each in six 384 plates treaded with concentration-dilution series of the COMBO drug plate (see Table 1 for drug order, each plate row is a dilution series in duplicates). Cells were imaged in three fluorescent colors (Red: Acin, Green:Tubulin & Blue:Nucleus, See image examples in top pannels). Individual cells were segmented, and for each cell an array of attributes was quantified. Attributes include cells and nuclei morphological and fluorescence intensities attributes and microtubules and actin fiber attributes (see Methods and *Extended data:* Table S2). Color coded Mahalanobis Scores (see *Extended data:* Figure S2) are presented here for each well in six plates for the five cell lines. A number of drugs with strong cellular effects are displayed (red rows), and indicate some cell-line dependence. Robot error effects were minimized in the analysis by rejecting outliers lying outside the robust PCA ellipsoids.

Five established cell lines; four derived from urinary-bladder cancers, and one from cervical carcinoma, were plated in 384-well plates at a density of 1000 cells per well. Following incubation for 24h, the cells were treated with serial dilutions of the COMBO plate drugs ^48^ (ten, three-fold dilutions, concentration range: (10-5×10 ^-4^)*GI _50_ , where GI _50_ is the concentration of the least sensitive line in the NCI60, see *Extended data:* Table S1 ^51^) and further incubated for 18h (see Methods). The cells were then fixed and labeled (see Methods). Images were acquired in these three fluorescent channels (see top panels, Figure 1), and analyzed as described in the Methods and in *Extended data:* Figure S1 ^51^. The analysis yielded, for each well, a list of segmented cells, each characterized by a “vector” of quantified features (‘attributes’).

Whole cell segmentation was commonly achieved by diffuse cytoplasmic staining (e.g. “cell mask”, Invitrogen), with enhanced nuclear concentration. Cytoplasmic fluorescence (due to the monomer fraction of tubulin and actin and even cell autofluorescence) plus the definition of the nucleus by DAPI (often excluding cytoskeletal proteins) provided highly reliable segmentation of individual cells, even in densely-packed cell islands. This approach enabled us to ‘free’ color channels for labeling cells for additional cellular markers of interest. In addition, the segmentation process allowed us to also calculate the total covered cell area which can report on cell spreading/proliferation/death during the time of treatment of each of the tested cell lines. Notably, although all lines are of epithelial origin, the RT4 cells (and to some extent HT1376) grow in islands even at low plating density, while TCCSUP and UMUC3 cells grow, primarily as isolated cells, and may be more susceptible to shrinking or contraction. For this reason, the analysis is based primarily on “per-cell” attributes.

**Figure 2.**
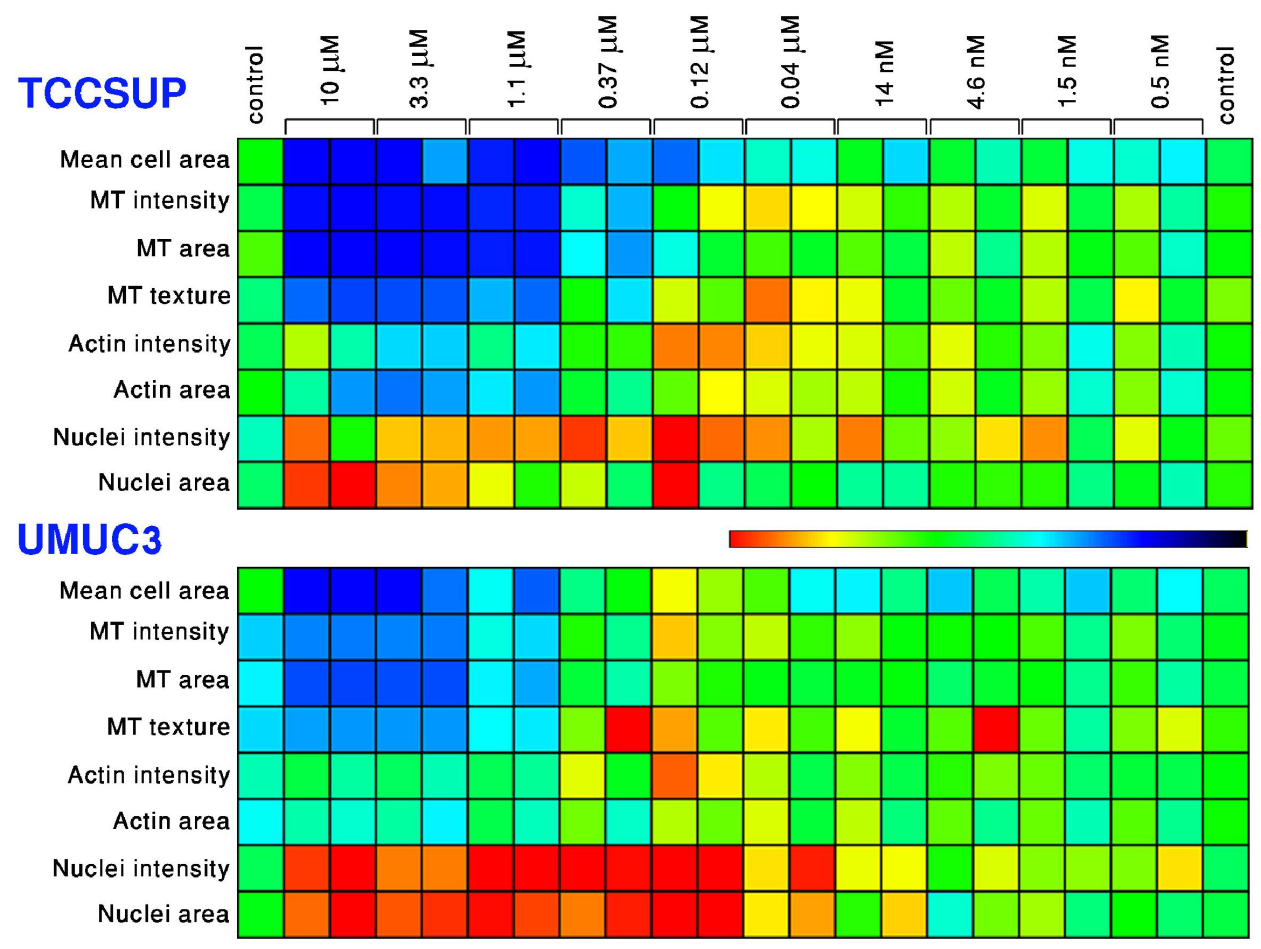
Different parameters report different response profiles. TCCSUP and UMUC3 cell lines were treated by nocodazole. Image analysis recorded nuclei, cells microtubule and actin-related scores. The response curves clearly depend on the attribute used to quantify the effects, and the strength of the effect depends on the cell line.

The profiling of drug responses based on a single attribute may be misleading, as demonstrated in Figure 2. Dose-response profiles of a single drug (nocodazole) is displayed by eight different attributes (cell area, MT intensity/ area/ texture, actin intensity/ area, nucleus intensity/ area) for two cell lines (TCCSUP, UMUC3). The results are displayed as a “virtual plate”. Various attributes display different responses: tubulin-related attributes in the first four rows indicate the disruption (Blue) of the microtubule network; attributes related to the actin cytoskeleton show little effect after 18 hours; nucleus-related attributes show an increase (Red) in DNA content per cell, possibly due to cell-cycle arrest.

In order to define an unbiased score that will accumulate drug-induced changes from multi-parametric characterization of cell properties, we have to consider three factors: (1) different attributes have different dimensions and scales; (2) the cell-by-cell variability of each attribute can be quite different; (3) it is not easy to select independent attributes. For example, cell content of a specific detected protein (either by expression of fluorescent derivative or by specific immune-labeling) is highly correlated with cell size. While average protein concentration accounts for this dependency, there are less obvious correlations, reflecting regulated cellular properties, which may become relaxed or tightened in response to drug treatments.

PCA is a well-established method for the characterization of multi-dimensional variability and correlations. We have applied PCA to the quantified attributes in the control wells to best fit a multi-dimensional hyper-ellipsoid, where elongated axes indicate correlated attributes. Using this hyper-ellipsoid, we defined a score based on Mahalanobis distances between each treated cell to the control cells (see Methods and *Extended data:* Figure S2 ^51^). This score balances the scales and variability of the measured attributes and accounts for correlations between them. In addition, the hyper-ellipsoid for each of the treated wells provides a multi-parametric scale for identifying outliers, created by technical artifacts such as bubbles, dead cells, cell clumps etc. Figure 1 shows the multi-parametric Mahalanobis scores for all the COMBO plate drugs applied to the five cell lines tested. The figure compiles 360 Gigabytes of image data (12GB/plate) imaged from 30 multi-well plates. The scores coded in spectral colors are displayed in the plate format that is also used as a “Graphic User Interface” (Plate GUI) showing, upon a mouse-click on a well, montages of the acquired images, outlined cell segments, as well as the full resolution image color components, and a wealth of statistical presentations and cell-by-cell numerical values, facilitating data mining within the large volume of information (see *Extended data:* Figure S1 ^51^).

The scores display differential responses to some of the drugs for some of the cell lines. Table 1 lists the AC _50_ values obtained for these drugs, as described in the Methods. It should be noted that defined as a “distance”, Mahalanobis scores are always positive. The multi-parametric score is a faithful reporter of changes in any of the measured attributes for all drugs that showed effects. Moreover, once an effect is detected by scoring large Mahalanobis distance to the control ellipsoid, the largest contributions of each of the attributes to the score identify the most significant attribute changes (see examples in Table 2). The plate scores for individual high contributors were qualitatively similar (though not identical) to the multi-parametric Mahalanobis scores, yet the latter present in one picture what can only be exhaustively reviewed by many single attribute scores. Altogether, this offers a fast and systematic method with internal standardization for navigating in multi-dimensional attribute space towards focusing on the cellular phenotype responding to perturbations.

**Figure 3.**
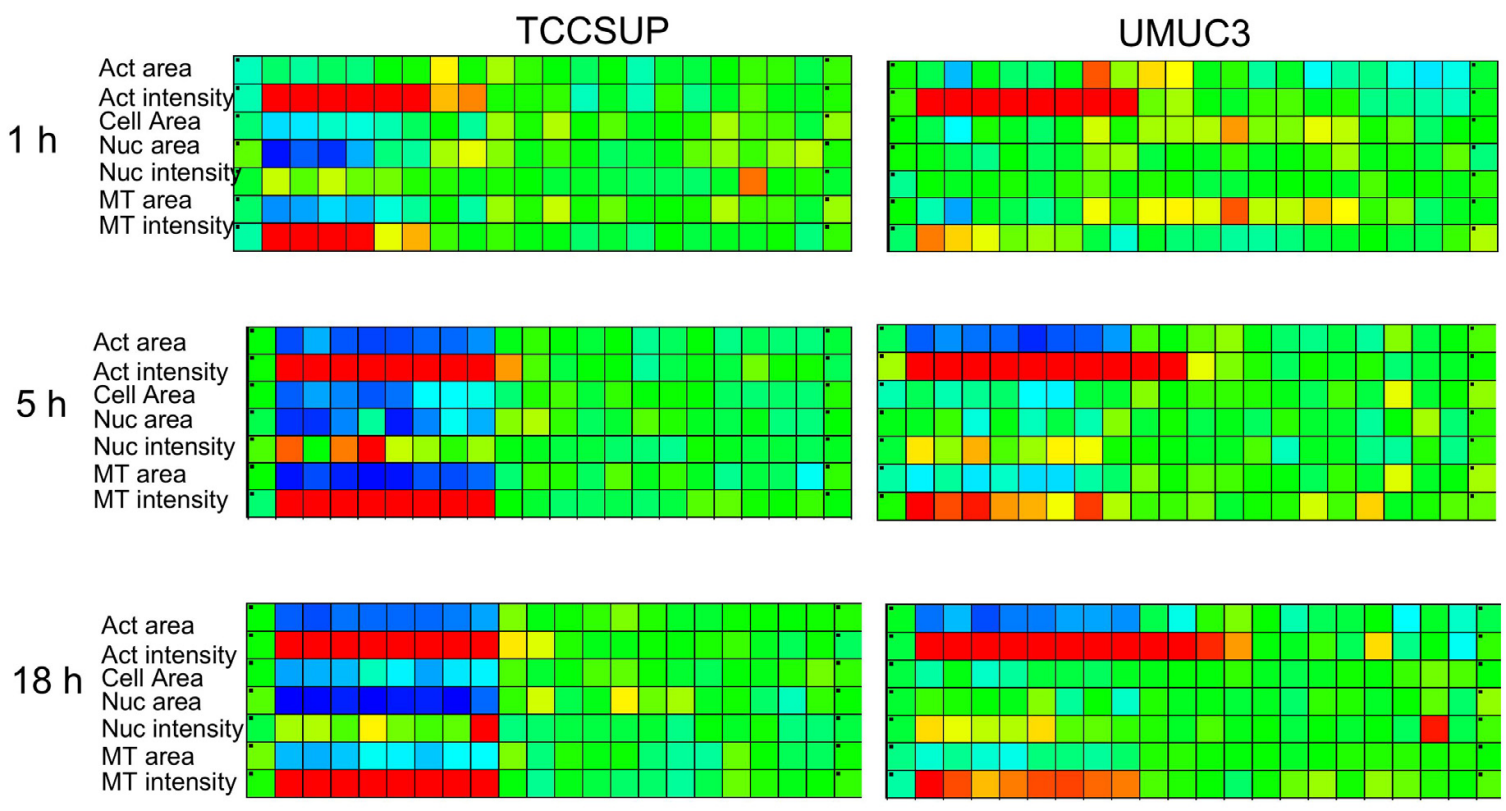
Comparison of short and long-term drug effects on attributes. Three plates were treated with 6 drugs for 3 times: 1, 5 & 18h (shown only Nocodazole for 2 cell lines for several attributes). The “virtual plate” displays in rows the different attributes evaluating cellular effects at different time points. Time-response fingerprinting based on a single attribute depends on the attribute chosen, and is not reliably reporting time-dependence of effects. For example, fast (minutes) disruption of microtubules, indirect effects on the actin filaments (an hour), changes in cell spreading, slower cell-cycle arrest (many hours) and cell death (days) are only reported by several time points.

Figure 3 depicts another important feature of cellular responses to drug treatments, namely, time dependence. The effect of drugs with specific molecular target is typically documented using reporter cells displaying target activation. However, the medical value of many drugs often stems from indirect effects. Multi-parametric cell-based measurements can probe both direct and indirect effects. Here the fast disruption of microtubules compared to the slower cell cycle arrest (depicted by DAPI-labeled DNA content) can be seen. Fingerprints of drug effects, based on an array of features or time-dependent of a single feature, suffer from different scales and variabilities of the measured features. The Mahalanobis-scaled fingerprints can include many features at several times, and present more balanced measures for analyses such as multiparametric similarity of drug effects.

**Table 2.** Mostly contributing attributes. Listed are examples for three drugs, Curcubitacin, Helenalin and Nocodazole, that strongly affected TCCSUP cells as seen in Figure 1 Plate 1 row 9, plate 3 row 11 and plate 6 row 12 respectively. (A) Curcubitacin-treated TCCSUP indicate rounding up compared to the control cells (Shrinking cells long axis, compare to control cells, the top of Figure 1), loss of actin stress fibers (strongly stained condensed actin), and longer, strongly labeled tubulin fibers. (B) Helenalin-treated cells show stronger total per-cell staining of actin & tubulin fibers, and also stronger DAPI staining, compatible with cell cycle arrest. (C) Nocodazole-treated cells indicate loss of tubulin fibers with no disturbance of actin filaments. All the above descriptions based on image visualization are compatible with the three mostly-contributing attributes to the Mahalanobis distance from controls, listed below. Other attributes have normalized Mahalanobis contributions of less than 0.2.

*(A) CURCUBITACIN Attribute by order of contribution to Score*			
Att. Name:	Cell_LongAxis	Tub_Fib Len	Act_Fib CelInt-Bck
Norm.Contr.Maha:	0.648291	0.347245	0.324186
*(B) HELENALIN Attribute by order of contribution to Score*			
Att. Name:	Tub AvgInt-Bck	Act CelInt-Bck	Nuc TotInt
Norm.Contr.Maha:	0.572778	0.521640	0.505878
*(C) NOCODAZOLE Attribute by order of contribution to Score*			
Att. Name:	Tub TotInt-Bck	Tub CelInt-Bck	Tub Len
Norm.Contr.Maha:	0.581077	0.430987	0.319133

**Figure 4.**
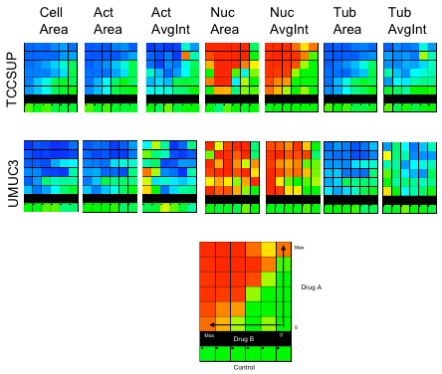
Drug combination essays. The combinations were selected from 20 "responsive" drugs ( Table 1). 16 two-drug combinations matrices were applied to two cell lines (TCCSUP & UMUC3). 5×5 matrices (10×5 wells including duplicates) + single-drug rows and columns + controls were prepared. 4 dual combinations were set in each plate, 8 plates total (4 for each cell line). Example of 7 single features response for the combination of Radicinin (rows) and Brasilin (columns) in concentrations of 4-0.25µM are displayed for the two cell lines (TOP). The data require preparation of larger matrices with more dilutions and repeats. Nevertheless we attempted to fit the Mahalanobis scores (BOTTOM) to Loewe or Bliss models (see Methods) but found no significant synergism.

In conclusion, multi-parametric analysis of cellular responses to drugs, visualized by high-definition light microscopy screening, allows departing from target-guided drug screening and build essays more sensitive to cell-level functional effects. In addition, since systematic screen of all drug combinations is not practical, selection based on multi-parametric analysis may increase the chance of identifying interactions between different cellular mechanisms favorably affected by drug combinations. In Figure 4 we show matrices of the responses of one combination (Radicinin and Brasilin) out of 16 binary combinations tested at five 2-fold dilutions around the “AC _50_” concentration of the single drug treatments. Differences between the two treated cell lines are seen for nuclear-related features. However, Loewe or Bliss model fitting to the Mahalanobis scores does not indicate synergism.

## Discussion

Images recording cell, nuclear and cytoskeleton structures were analyzed for five cell lines treated with the COMBO drug library and revealed differential responses. Cell morphology and tagged protein intensity and distribution attributes depicted cellular responses as reported by various features. The transition with time from direct and specific effects on the drug target to indirect and distributed responses manifest the advantages of high-resolution imaging for characterization of cell-level responses by multi-parametric scores. Identification of the features mostly affected by each treatment help guide to relevant cellular functions mediating these responses.

Both basic biological research and biomedical challenges need high-throughput technologies to address the complexity of cellular mechanisms. The information-content in high-throughput experiments, initially confined to biochemical assays reporting a specific target protein, is extending to detailed quantitative sub-cellular characterization, classically the basis for understanding cellular functions. This work developed analysis for high-resolution (recording cytoskeletal fibers) multi-color cellular images from large-scale screens, and display of the results in a digestible form with cell-informatics mining capability. Multi-parametric quantification of cellular effects offers a method to identify cellular responses to drugs without selecting in advance a specific drug targets as a reporter. Effective AC _50_ obtained from multi-parametric analysis integrate many cell-level responses and is therefore less essay-biased then measurements based on single-attribute analysis. Scores based on Mahalanobis distance offer standardized measures of responses in comparison to controls.

Perturbations to living systems, even when the treatment is directed towards a highly specific target with well-defined short-term effects, cause at longer times distributed cellular responses. Characterization of the time-dependence of the direct and indirect responses to drugs using multi-parametric analysis contain causal information to help dissect mechanisms, and may provide rational basis for optimization of temporal schedules for drug administration, already recognized valuable in chemotherapy ^51^.

Multi-component drug combinations are commonly used in chemotherapy, AIDS and also in antibiotic treatments to fight development of resistant bacteria clones. The effectiveness of such cocktails is often a result of interactions at the physiological level, and was optimized through slow clinical trials. Redundancies and multi-functionalities in molecular networks mediating cellular mechanisms ^59– 62^ and the multi-genetic nature of diseases such as cancer, with abnormal protein interactions and modified cellular mechanisms, suggest a potential for the discovery and optimization of multiple-perturbation approaches using cell preparations. Many classical drug targets are shared by the disease phenotypes and the normal ones, causing undesired side effects at the potent dozes. Multiple-drug treatments offer degrees of freedom to optimize synergistic interference with a specific mechanism, preferably more effective in the diseased version and in a given cell lineage, with reduced side effects due to lower doses of each individual drugs in the mixture and minimize compensation by alternative pathways. The benefits of compounds with lower affinity and multiple targets is therefore re-evaluated due to their potential in combinatorial therapies ^63– 67^.

The advancement in our understanding of the molecular mechanisms of cellular functions serves as a basis for rational design of drugs. Combinatorial drug effect profiling is in fact a tool to probe normal network structures ^60– 62^. However, first principles quantitative modeling of complex cellular networks depends on many and not readily measurable parameters. Cell-based high-content drug response profiling is therefore a practical method to study multiple perturbations, and to optimize mixtures with advantageous responses. Cell-based assays also open possibilities for patient-specific profiling of abnormal pathways and personal optimization of treatments.

## Data availability

### Underlying data

The raw data includes 10 Terabytes of images and are therefore too large to share. The images are kept on storage disks as OME-TIFF files with .xml headers metadata for each image. Individuals wishing to access this data can apply to the corresponding author in order to obtain the files. In this situation, these files will be uploaded to and available from the Weizmann Institute of Science website. Example images for one plate are provided in the *Extended data* ^67^.

### Extended data

Figshare: Multi-parametric characterization of drug effects on cells, https://doi.org/10.6084/m9.figshare.12981518.v2.

This project contains the following extended data:

- Table S1. The order of transfer from the COMBO 96-well plates and the COMBO plate handout.
- Table S2. The list of attributes calculated for each cell.
- Figure S1. Analysis Flow Diagram.
- Figure S2. Mahalanobis distance score.

Figshare: Multi-parametric characterization... Original Data, https://doi.org/10.6084/m9.figshare.12981875.v1 ^55^.

- Analysis scripts
- Example images for one plate
- Analyzed data for the example images (Excel file)

Data are available under the terms of the Creative Commons Zero "No rights reserved" data waiver (CC0 1.0 Public domain dedication).

## Notes

^1^RT4 cells were received to the lab in November 2006 from the ATCC. RT4 cells are much smaller than HeLa and have different morphology. We could clearly see that there was no HeLa contamination in our RT4 cells.
