# Supplementary material for: Multi-parametric characterization of drug effects on cells
Source: F1000Res. 2021 Jan 21;9:ISF-1199. Originally published 2020 Oct 5. [Version 2] doi: 10.12688/f1000research.26254.2 (PMC7737707; doi:10.12688/f1000research.26254.2)
Supplement: Peer review history

# Peer review: Reviewer response for version 1

**DOI:** 10.5256/f1000research.28977.r75113

**Published:** 2020-12-14

**Authors:** Robert Murphy

**Affiliations:** 1 Computational Biology Department, School of Computer Science, Carnegie Mellon University, Pittsburgh, PA, USA

This manuscript describes high throughput microscopy measurement and analysis of the effects of drugs using multiple fluorescent probes. While the study has been carefully done and the results for particular drugs may be of interest to researchers, the manuscript does not represent a significant advance either in experimental approach or in subsequent analysis. The use of multiple simultaneous fluorescent probes is widespread in biomedical research, including in drug development. For example, the work on cell painting described by Bray *et al.* is has been widely cited and used. ^1^ The use of similar multivariate statistical methods for analysis of the signals from those probes is also routine.

Is the rationale for developing the new method (or application) clearly explained?

Yes

Is the description of the method technically sound?

Yes

Are the conclusions about the method and its performance adequately supported by the findings presented in the article?

Partly

If any results are presented, are all the source data underlying the results available to ensure full reproducibility?

No

Are sufficient details provided to allow replication of the method development and its use by others?

Yes

Reviewer Expertise:

computational cell biology, biological image analysis and modeling

I confirm that I have read this submission and believe that I have an appropriate level of expertise to confirm that it is of an acceptable scientific standard.

## References

[1] : Cell Painting, a high-content image-based assay for morphological profiling using multiplexed fluorescent dyes. *Nat Protoc*.11(9) : 10.1038/nprot.2016.105 1757-74 10.1038/nprot.2016.105 27560178PMC5223290

# Response

**Published:** 2020-12-19

**Authors:** Zvi Kam

**Affiliations:** Weizmann Institute of Science, Israel

We believe that the ability to detect drug effects on cells and relate them, in an unbiased fashion to quantitative attributes extracted from the images is a key feature that is missing when screens are directed towards identification of drugs or genes related to predetermined functional or morphological effects. The unbiased approach provided by Mahalanobis score and the identification of "most contributing attributes" to score differences from controls may reveal unexpected results in laborious screen preparations, independently of the initial aims of the work.

# Peer review: Reviewer response for version 1

**DOI:** 10.5256/f1000research.28977.r72645

**Published:** 2020-10-26

**Authors:** John Lock

**Affiliations:** 1 School of Medical Sciences, University of New South Wales, Sydney, Australia

This article delineates a novel strategy and accompanying analytical platform to assess and compare the complex, multiparametric effects of drug treatments on cell populations (at the single cell level). The platform, described in detail, couples cell phenotype (fluorescence) imaging and an integrated quantitative image analysis and statistical data analysis/visualisation capability that enable rapid exploration of cellular states/responses across multiple scales of the data hierarchy.

 Focused on data capturing the level of single cell phenotypes measured through imaging with subcellular resolution, this article captures an analytical strategy that is relevant to drug discovery, but also to fundamental analysis of cellular processes, their underlying mechanisms, and potentially, diagnostic assessment/identification of defective processes in disease states. 

 Using the NCI COMBO drug library as a basis for analysis, the authors detail the experimental, imaging, and image analysis process, but reserve particular focus for the methods of drug effect analysis, primarily relying on the Mahalanobis distance. This measure captures a compact representation of drug effect strengths relative to control variation, whilst also embedding information about the (relative weights) of specific cellular features that capture/comprise the effects of any given perturbation (drug). Mahalanobis distance thus emerges as a powerful tool to assess, compare, sort, and visualise drug effects, especially when compared (as the authors show) with single feature responses, which are far less systematic in their indications. 

 The authors also highlight the strengths of their approach for identifying complex temporal dynamics of drug response, i.e. how changes in different phenotypic features emerge at different times after treatment. Reflecting the way that even highly specific initial perturbation impacts are propagated outward over time within cellular information processing and responses networks, this is an important additional dimension that is precisely addressed using the authors' analytical strategy.

    

 Importantly, due to the very large scale of the datasets described, the authors indicate (reasonably so) that it is not feasible to share the entire dataset online. However, they indicate that, on request, components, or the entirety of the dataset can be accessed based on a request to the corresponding author. I interpret this as constituting data availability *in practice*.

Is the rationale for developing the new method (or application) clearly explained?

Yes

Is the description of the method technically sound?

Yes

Are the conclusions about the method and its performance adequately supported by the findings presented in the article?

Yes

If any results are presented, are all the source data underlying the results available to ensure full reproducibility?

Yes

Are sufficient details provided to allow replication of the method development and its use by others?

Yes

Reviewer Expertise:

Systems Microscopy of cancer cell biology, including signalling, cytoskeletal regulation, adhesion and migration biology. Phenotypic drug screening. Development of image analysis, statistical analysis, and data visualisation tools for quantitative single cell analysis based on imaging data.

I confirm that I have read this submission and believe that I have an appropriate level of expertise to confirm that it is of an acceptable scientific standard.
